# Effect of type 2 diabetes on liver images of GD-EOB-DTPA-enhanced MRI during the hepatobiliary phase

**DOI:** 10.1038/s41598-023-27730-0

**Published:** 2023-01-11

**Authors:** Wen-Yu Zhang, Hao-Yang Sun, Wen-Long Zhang, Rui Feng

**Affiliations:** 1 Department of Radiology, Shandong Cancer Hospital and Institute, Shandong First Medical University and Shandong Academy of Medical Sciences, Jinan, People’s Republic of China; 2 Department of Radiology, Affiliated Hospital of Shandong University of Traditional Chinese Medicine, No.16369 Jingshi Road, Jinan, 250014 Shandong People’s Republic of China

**Keywords:** Magnetic resonance imaging, Diabetes

## Abstract

To analyze alterations of the liver appearance during the hepatobiliary phase of individuals with type 2 diabetes who are receiving gadolinium ethoxybenzyl diethylenetriamine pentaacetic acid (Gd-EOB-DTPA) enhanced magnetic resonance imaging (MRI). Fifty-seven individuals who received Gd-EOB-DTPA-enhanced MRI and had normal liver and renal function but did not have (control group) or have type 2 diabetes (observation group) were retrospectively included in this study. The liver enhancement ratio (LER) and contrast between liver parenchyma and portal vein (LPC) were calculated from hepatobiliary phase images. Utilizing liver to kidney signal intensity, signs of the biliary system, and signs of the portal vein, a functional liver imaging score (FLIS) was calculated. Wilcoxon rank-sum test was used to assess the between-group differences in LER, LPC, and FLIS. FLIS constituent ratios between the two groups were tested using the *χ*^*2*^ test. The effectiveness of LER, LPC, and FLIS for identifying type 2 diabetes was assessed by receiver operating characteristic curves (ROCs). The interobserver consistency of FLIS was evaluated using the intraclass correlation coefficients. The observation group’s LER and LPC were lower than the control group. The constituent ratio of the FLIS score (liver to kidney signal intensity, *p* = 0.011) showed a significant between-group difference. According to ROCs, LER and LPC were associated with the identification of type 2 diabetes. LER = 0.54 and LPC = 1.46 were the optimal cutoff for identifying type 2 diabetes, respectively. FLIS demonstrated excellent inter-reader agreement. The relative signal intensity of the liver during the hepatobiliary phase is decreased in patients with type 2 diabetes. This should be considered when individuals with type 2 diabetes undergo Gd-EOB-DTPA-enhanced MRI to avoid misdiagnoses, such as small hepatocellular carcinoma or abnormal liver function.

## Introduction

Gadolinium ethoxybenzyl diethylenetriamine pentaacetic acid (Gd-EOB-DTPA) is used as a contrast agent for magnetic resonance imaging (MRI) examination of the liver. Gd-EOB-DTPA reflects anatomical information, blood supply, and liver function^1^ and is widely used for diagnosing liver tumors, evaluating patient’s liver function in cirrhosis, predicting liver failure after liver tumor resection, and assessing postoperative biliary complications^2–4^. Organic anion-transporting polypeptides (OATPs) located in the liver membrane take Gd-EOB-DTPA into hepatocytes, and multidrug resistance-associated protein 2 subsequently excrete it into the biliary tract^5^. Specific food components, drugs, malignancies^6–8^, chronic liver diseases, nonalcoholic fatty liver diseases, liver fibrosis, and inflammation can influence the function and expression of OATPs and alter their pharmacokinetics^9–11^. In type 2 diabetic mice, OATPs expression in hepatocytes is reduced, and Gd-EOB-DTPA uptake by hepatocytes is diminished, leading to a secondary decrease in hepatic signal intensity^12^.

The number of individuals with type 2 diabetes is increasing worldwide^13^; however, changes in hepatobiliary imaging attributable to type 2 diabetes are poorly studied. We evaluated the effect of type 2 diabetes on hepatobiliary images obtained from individuals who received Gd-EOB-DTPA-enhanced MRI.

## Materials and methods

### Patients

The local institutional ethics review board (Ethics Committee of the Affiliated Cancer Hospital of Shandong First Medical University) approved our retrospective study. All experimental protocols were performed in accordance with the relevant guidelines and regulations of this committee and the Declaration of Helsinki (2000). The Ethics Committee of the Affiliated Cancer Hospital of Shandong First Medical University approved the informed consent waiver due to a retrospective study.

**Figure 1:**
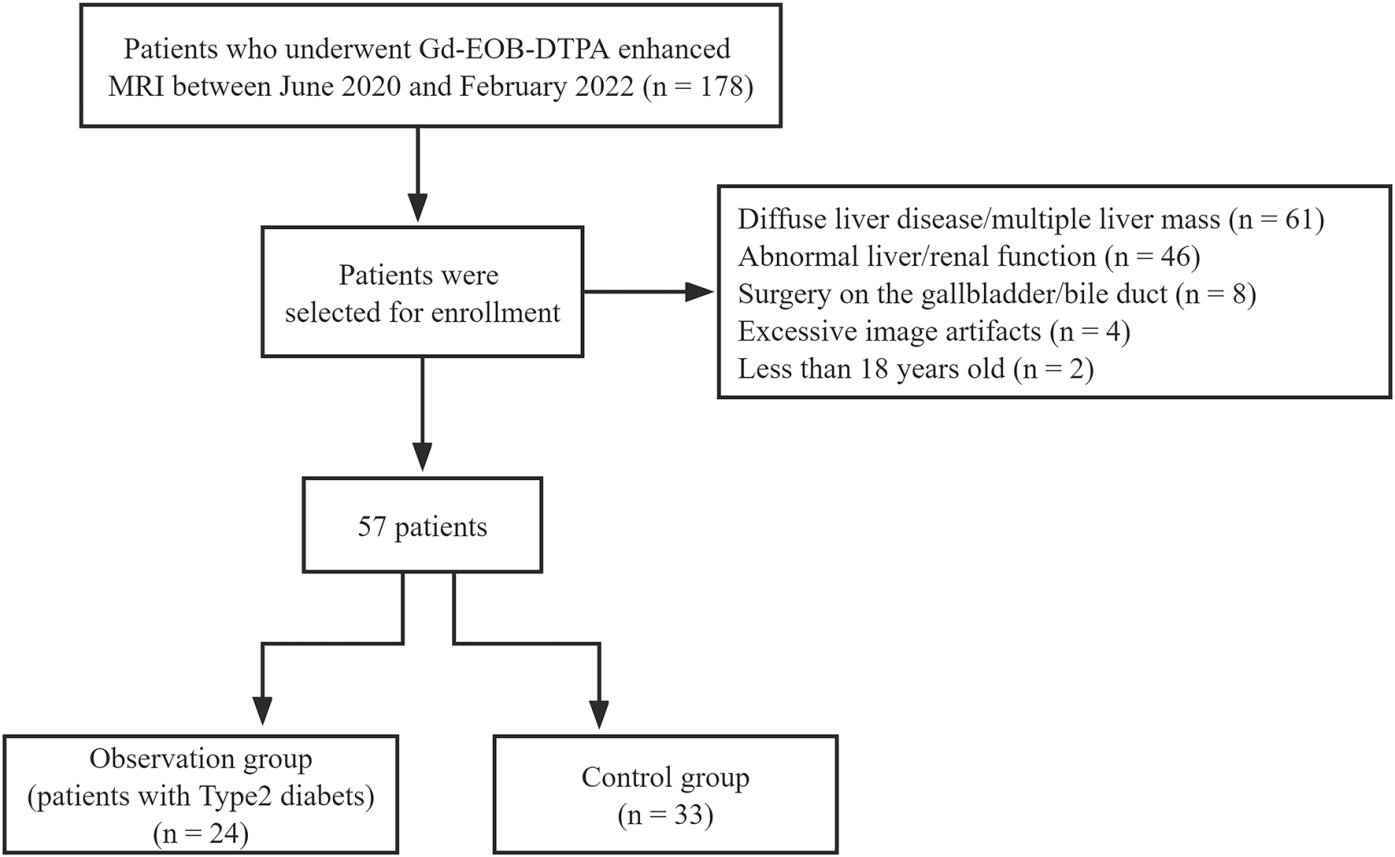
Flow diagram of the study population. Gd-EOB-DTPA, gadolinium ethoxybenzyl diethylenetriamine pentaacetic acid; MRI, magnetic resonance imaging.

The clinical data of 178 individuals who received Gd-EOB-DTPA-enhanced MRI from June 2020 to February 2022 were retrospectively analyzed. We excluded patients with diffuse liver disease (fatty liver, viral hepatitis, liver fibrosis, cirrhosis, etc.) or multiple (> 5) liver masses; abnormal liver or renal function; who underwent cholecystectomy or nephrectomy; whose images featured excessive artifacts; and who were younger than 18 years (Fig. 1). The above exclusion was based on the patient’s past medical history and the relevant routine examination results after admission, such as serum creatinine, blood urea nitrogen, cystatin C, β2-microglobulin, uric acid, biochemical liver test, hepatitis virus nucleic acid test, antigen–antibody tests for various viral hepatitis, B ultrasound, or MRI, etc. The results of those tests should be negative or have very few minor biochemical abnormalities. None of the subjects had taken any drugs or foods known to affect MRI results (e.g., statins, rifampicin, quercetin, or certain antineoplastic drugs) for at least 2 months before undergoing Gd-EOB-DTPA-enhanced MRI. Participants’ data—including age, sex, body mass index, admission diagnosis, and diagnosis of type 2 diabetes—were recorded. Type 2 diabetes was diagnosed according to the 1999 World Health Organization diagnostic criteria.

### Imaging techniques

This study used 3.0 T MRI (MAGNETOM Skyra 3.0 T, Siemens, Germany) to examine all subjects. Standard 16-channel phased-array body coil and 32-channel phased-array spinal coil were applied to the coils. T1-weighted images (T1WI) were captured using rapid dynamic enhanced imaging sequences before and after contrast injection. The protocol was as follows: TR/TE, 3.97 ms/1.29 ms; slice thickness, 6 mm; flip angle, 9°; field of view, 380 mm × 320 mm; Matrix: 360 × 100%. Gd-EOB-DTPA (Primovist/Eovist, Bayer Healthcare, Germany) was administered intravenously (0.025 mmol/kg, 1.0 mL/s) and was then flushed with 20 mL of saline. Triple arterial phase, portal venous, transitional, and hepatobiliary phase images (20–35 s, 60 s, 300 s, and 20 min after injection) were acquired with a single breath-hold approach. After scanning, all imaging data were uploaded and evaluated.

### Image analysis

One of the radiologists (R.F., with five years of clinical experience) collected clinical data from all observed subjects. Two radiologists (H.Y.S. and W.Y.Z., with more than ten years of clinical experience) independently reviewed the images. They were unaware of the clinical information of each patient.

The region of interest (ROI) was plotted on the pre-enhancement T1WI and hepatobiliary phase images, and the signal intensity was measured. ROI was taken for each of the left lateral liver lobe, left medial liver lobe, right anterior liver lobe, and right posterior liver lobe. The ROI area of approximately 2.50 cm^2^ was created for each lobe, and focal lesions, blood vessels, and bile ducts were avoided. SI_L0_ and SI_L1_ represented the liver signal intensity before and after enhancement. The hepatic portal signal intensity (SI_PV_) was measured at the porta hepatis level on the hepatobiliary phase images. SI_PV_ was the signal intensity of the ROI set within the portal vein. The diameter of the circular ROI was slightly smaller than the maximum transverse diameter of the portal vein. The average value was calculated by continuously selecting three slices.

The liver enhancement ratio (LER) and the contrast between liver parenchyma and portal vein (LPC) were calculated as follows^14^:

$$ {\text{LER}} = \left( {{\text{SI}}_{{{\text{L1}}}} - {\text{ SI}}_{{{\text{L}}0}} } \right)/{\text{SI}}_{{{\text{L1}}}} ;{\text{ LPC}} = {\text{SI}}_{{{\text{L1}}}} /{\text{SI}}_{{{\text{PV}}}} $$

**Figure 2:**
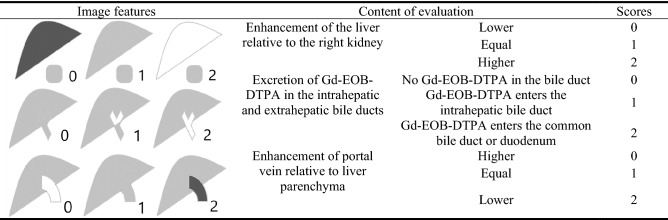
FLIS score descriptions. FLIS, Functional Liver Imaging Score; Gd-EOB-DTPA, gadolinium ethoxybenzyl diethylenetriamine pentaacetic acid.

The Functional Liver Imaging Score (FLIS) was determined according to the hepatobiliary phase images. Based on the FLIS classification scheme (Fig. 2)^2,15^, three performance scores/sub-items (consisting of independent scores for the liver to kidney signal intensity, signs of the biliary system, and signs of the portal vein) were calculated. Each sub-item was assigned a score of 0, 1, or 2. The FLIS, which ranged from 0 to 6, was the total of the three performance scores. The scores of the two radiologists were recorded separately, and then their scores were discussed to reach a consensus on the final scores.

### Statistical analysis

LER, LPC, and FLIS (including the three sub-items) were presented as mean ± standard deviation or median (interquartile range) according to whether they fit into a normal distribution. Between-group differences were compared using the Wilcoxon rank-sum test. To further analyze group differences in the three sub-items of the FLIS, the constituent ratios of the sub-items scores were described as frequencies (%), and between-group differences were tested by the *χ*^*2*^ test. Receiver operating characteristic curves (ROCs) was used to evaluate whether LER, LPC, and FLIS could help to identify type 2 diabetes. The interrater consistency of FLIS was evaluated using the intraclass correlation coefficients (ICCs). SPSS Statistics (version 25, IBM) was used for all statistical calculations. All tests were two-sided, and significance was determined by *p* values < 0.05.

### Ethical approval

All procedures performed in studies involving human participants were in accordance with the ethical standards of the institutional research committee and the 1964 Declaration of Helsinki and its later amendments or comparable ethical standards. This retrospective study was approved by Institutional Review Board.

### Consent to participate

Due to its retrospective design, written informed consent was waived by the Institutional Review Board.

## Results

### Characteristics of the study population

A total of 57 patients were enrolled in the study, including 31 males and 26 females. The average age of the subjects was (58.68 ± 12.13) years old, ranging from 22 to 79. All subjects were selected according to the exclusion criteria mentioned above, which ensured that the study results were not interfered with by other conditions such as abnormal liver or kidney function as much as possible.

The observation group consisted of patients with type 2 diabetes [n = 24; mean age, (62.63 ± 9.39) years; the course of type 2 diabetes, (3–84) months; 3 patients were treated with insulin injection; 21 patients were treated with oral drugs; fasting blood glucose was controlled within the normal range in all patients, and the glycated hemoglobin level in all patients was less than 7% (HbA1c < 7%)]. There were 2 cases of hepatocellular carcinoma, 2 cases of intrahepatic cholangiocarcinoma, 1 case of hepatic hemangioma, and 19 cases of hepatic metastases in the observation group (primary tumors included colon cancer, n = 12; rectal cancer, n = 3; lung cancer, n = 2; breast cancer, n = 1; pancreatic cancer, n = 1). The control group included patients without diabetes [n = 33; mean age, (55.82 ± 13.18) years]. The control group included 4 cases of hepatocellular carcinoma, 1 case of intrahepatic cholangiocarcinoma, and 28 cases of hepatic metastases (primary tumors included colon cancer, n = 16; rectal cancer, n = 2; esophageal cancer, n = 2; lung cancer, n = 3; breast cancer, n = 1; pancreatic cancer, n = 1; malignant melanoma, n = 1; ovarian cancer, n = 2). There were no statistically significant differences in age, sex, or body mass index between the two groups (Table 1).

**Table 1:** Clinical characteristics of the patients.

Contents	Observation group	Control group	*p* value
Age (years)	62.63 ± 9.39	55.82 ± 13.18	0.071
Men (%)	13 (54.2%)	18 (54.5%)	0.98
Women (%)	11 (45.8%)	15 (45.5%)	
Body mass index	19.76 (3.03)*	21.30 (5.72)	0.087

*Data that did not follow a normal distribution were presented as median (interquartile range).

### LER, LPC, and FLIS

**Figure 3:**
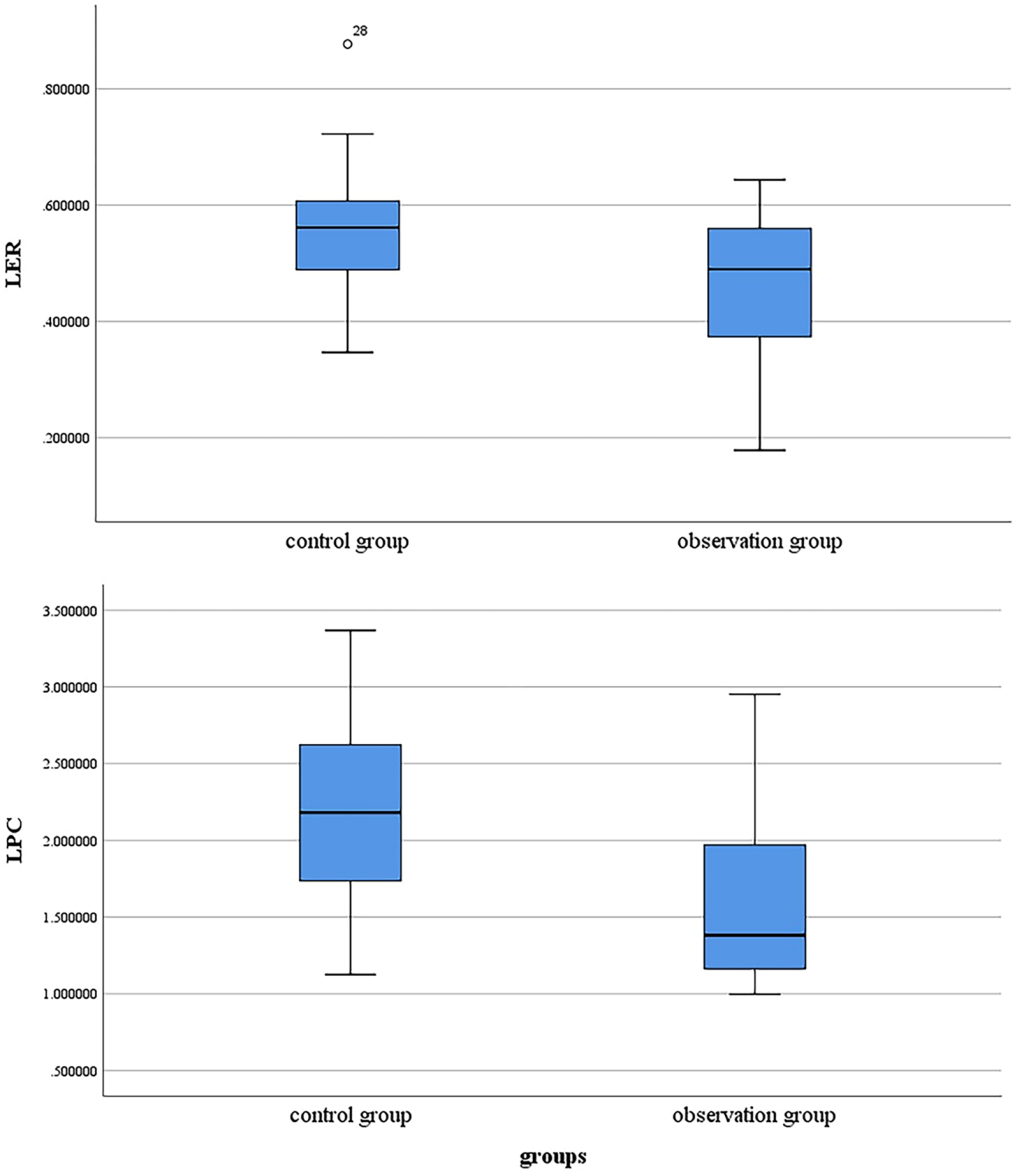
LER and LPC box plots for the two groups. LER and LPC were significantly lower in the observation than in the control group (LER,* p* = 0.008; LPC, *p* = 0.002). LER, liver enhancement ratio; LPC, contrast between liver parenchyma and portal vein.

There were significant between-group differences in LER [observation group 0.49 (0.21) vs. control group 0.56 (0.13), *p* = 0.008), and LPC [observation group 1.38 (0.88) vs. control group 2.18 (1.02), *p* = 0.002]. These results are presented in Table 2 and Fig. 3.

**Table 2:** Patients’ imaging examination results.

Contents	Observation group	Control group	*p* value
LER	0.49 (0.21)*	0.56 (0.13)	0.008
LPC	1.38 (0.88)	2.18 (1.02)	0.002
FLIS	6 (1)	6 (0)	0.067
Liver to kidney signal intensity	2 (1)	2 (0)	0.011
Signs of the biliary system	2 (0)	2 (0)	0.49
Signs of the portal vein	2 (0)	2 (0)	0.38

*Data that did not follow a normal distribution were presented as median (interquartile range).

*LER* Liver enhancement ratio, *LPC* Contrast between liver parenchyma and portal vein, *FLIS* Functional liver imaging score.

**Figure 4:**
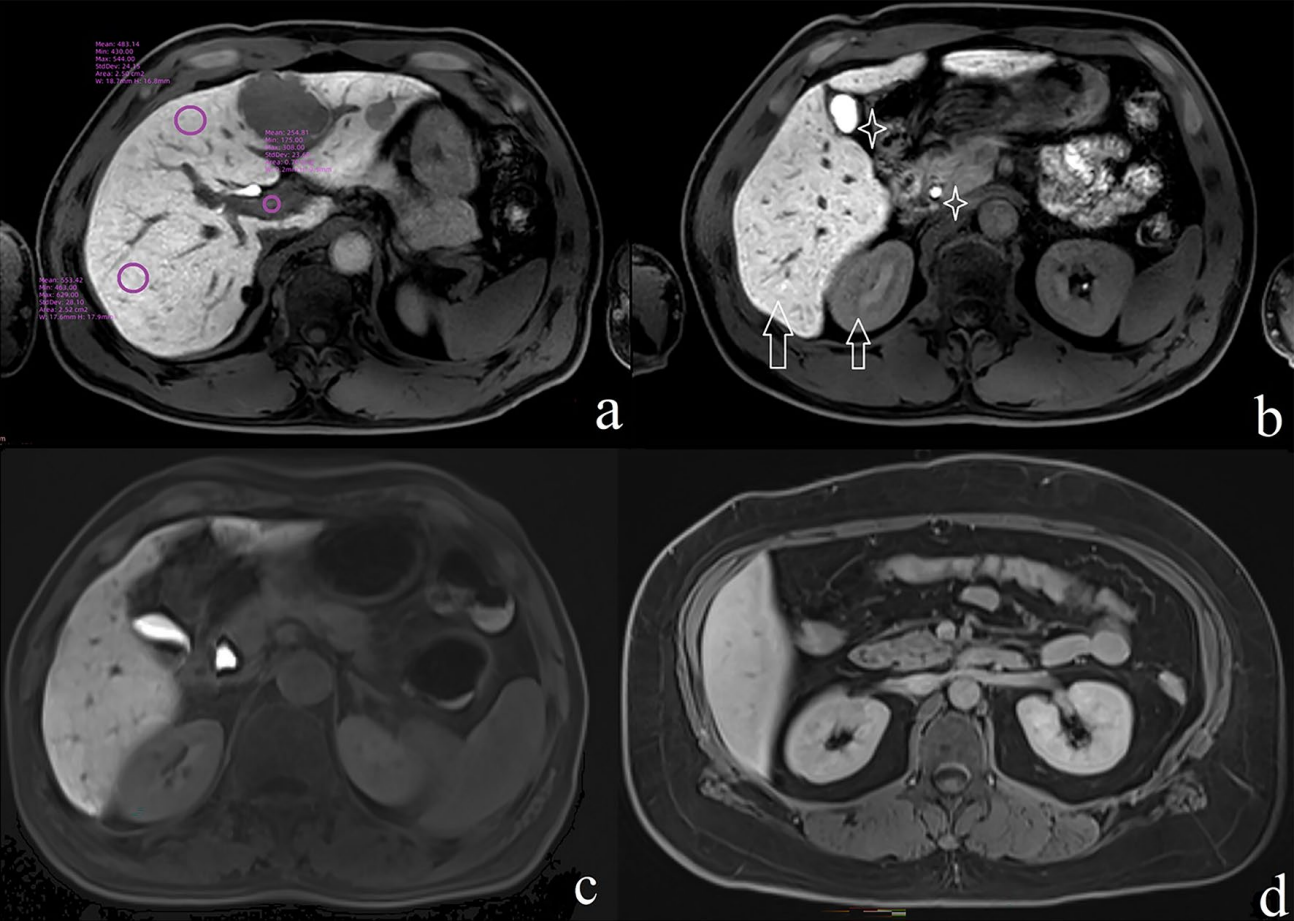
(**a**, **b**) The hepatobiliary phase images of a 45-year-old male with hepatic metastases but no type 2 diabetes (control group) who received Gd-EOB-DTPA-enhanced liver MRI. (**a**) The circles indicate signal intensity measurements in the liver parenchyma and portal vein; enhancement of portal vein relative to liver parenchyma (score 2) is shown. (**b**) Excretion of Gd-EOB-DTPA in the gallbladder and extrahepatic bile ducts (white stars, score 2) and enhancement of the liver relative to the right kidney (white arrow, score 2) are shown. In this case, the FLIS score was 6. (**c**) The hepatobiliary phase images of a 50-year-old female with hepatic metastases and type 2 diabetes (observational group). Gd-EOB-DTPA excretion in the extrahepatic bile ducts (score 2) and enhancement of the liver relative to the right kidney (score 2) are shown; however, her liver signal intensity is lower than the previously discussed male case. (**d**) The hepatobiliary phase images of a 59-year-old female with hepatic metastases and type 2 diabetes. Enhancement of the liver relative to the right kidney (score 1). Gd-EOB-DTPA, gadolinium ethoxybenzyl diethylenetriamine pentaacetic acid; MRI, magnetic resonance imaging.

There was no significant between-group difference in FLIS [observation group 6 (1) and control group 6 (0) (*p* = 0.067)]. That was, the sum scores of the three sub-items of the FLIS (liver to kidney signal intensity, signs of the biliary system, and signs of the portal vein) were not statistically different between the two groups. Further statistical analysis of the three parameters showed that the scores of the liver to kidney signal intensity had statistically significant differences between groups [observation group 2 (1) vs. control group 2 (0), *p* = 0.011, Table 2]. And the constituent ratio of the scores of the liver to kidney signal intensity (score 0, 1, 2) had statistically significant differences between groups (*p* = 0.011, Table 3, Fig. 4). However, the scores and the constituent ratios in signs of the biliary system and signs of the portal vein between the two groups were not significant (*p* > 0.05, Table 2, 3).

**Table 3:** Between-group differences in the three component scores of the FLIS.

Components of the FLIS	Score 0	Score 1	Score 2	*p* value
Liver to kidney signal intensity				
Observation group	0 (0%)	10 (41.7%)	14 (58.3%)	0.011
Control group	0 (0%)	4 (12.1%)	29 (87.9%)	
Signs of the biliary system				
Observation group	1 (4.2%)	0 (0%)	23 (95.8%)	1.00
Control group	2 (6.1%)	1 (3.0%)	30 (90.9%)	
Signs of the portal vein				
Observation group	0 (0%)	2 (8.3%)	22 (91.7%)	0.78
Control group	0 (0%)	1 (3.0%)	32 (97.0%)	

*FLIS* Functional liver imaging score.

**Figure 5:**
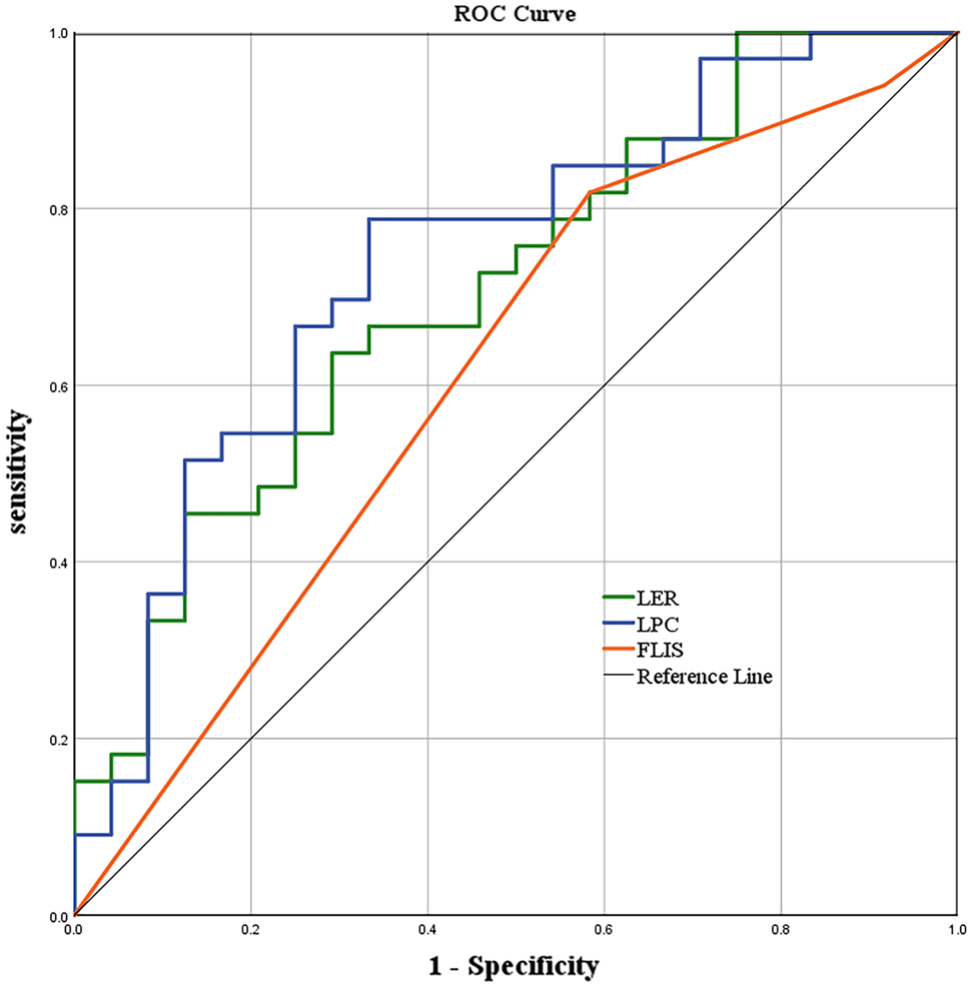
Receiver operating characteristic curves evaluating the effectiveness of LER, LPC, and FLIS in the differential diagnosis of type 2 diabetes. LER, liver enhancement ratio; LPC, contrast between liver parenchyma and portal vein; FLIS, Functional Liver Imaging Score.

ROC analysis for LER, LPC, and FLIS in differentiating type 2 diabetes are shown in Table 4 and Fig. 5. LER and LPC were associated with the identification of type 2 diabetes. FLIS was unable to identify type 2 diabetes (*p* = 0.15).

**Table 4:** Efficacy of LER, LPC, and FLIS for differentiating type 2 diabetes.

	AUC (95% CI)	Optimal criterion	*p* value	Sensitivity (%)	Specificity (%)
LER	0.71 (0.57, 0.84)	0.54	0.008	63.6	70.8
LPC	0.74 (0.61, 0.88)	1.46	0.002	78.8	66.7
FLIS	0.61 (0.46, 0.76)	5.5	0.15	81.8	41.7

*AUC* Area under the curve, *95% CI* 95% confidence interval, *LER* Liver enhancement ratio, *LPC* Contrast between liver parenchyma and portal vein, *FLIS* Functional liver imaging score.

### Interobserver agreement

ICCs (95% CI) for the FLIS, liver to kidney signal intensity, signs of the biliary system, and signs of the portal vein interrater agreement were 0.95 (0.92, 0.97), 0.84 (0.74, 0.90), 0.86 (0.78, 0.92), and 0.89 (0.81, 0.93), respectively.

In addition, after this MRI examination, we reviewed the follow-up liver and kidney examination results of all subjects during hospitalization, and no liver or kidney function damage was found related to this examination.

## Discussion

Gd-EOB-DTPA is used to measure liver function and diagnose multiple liver diseases^16–19^. Patients with severe or uncontrolled diabetes showed lower absorption of Gd-EOB-DTPA into the hepatocytes^20^. We investigated how the relative signal intensity of the liver on Gd-EOB-DTPA-enhanced MRI was altered in patients with type 2 diabetes compared with controls.

The hepatocytes’ capacity to absorb Gd-EOB-DTPA and liver function are reflected by LER and LPC^14,18,21^. Radiologists can diagnose liver dysfunction due to various causes using the visual scoring scheme FLIS based on subjective MRI findings^15^. LER, LPC, and FLIS do not require complex calculations or specific software and are not affected by magnetic resonance field strength, so they can be very convenient for the non-invasive study of liver function^22^.

We observed lower LER and LPC values in individuals with type 2 diabetes compared to controls. The findings matched those of a recent study^20^, but the severity of diabetes in our observation group (HbA1c < 7%) was lower than in their counterparts (HbA1c ≥ 8.4%), which was an essential difference between the two studies. This implied that common type 2 diabetes could lead to changes in liver images during the hepatobiliary phase. We speculated that the reduced signal intensity of liver parenchyma causes lower LER and LPC, given that our inclusion/exclusion criteria excluded interference from conditions like fatty liver, hepatitis, cirrhosis, biliary obstruction, or other liver diseases. We supposed that these results might indirectly reflect the reduced expression of OATPs in diabetic patients, similar to the mouse model^12^. According to ROC analysis, LPC and LER were helpful in the differentiation of type 2 diabetes (provided that other liver diseases mentioned in this article were excluded).

Although the between-group difference in the FLIS score as the sum score of three sub-items was insignificant, we found a significant decrease in the observation group's liver to kidney signal intensity score: the proportion of patients with a score of 1 was higher in the observation group. This could have occurred as a result of reduced liver enhancement. Additionally, the renal signal intensity might change accordingly. About half of the Gd-EOB-DTPA is excreted through the urinary system. Previous studies have confirmed that when liver dysfunction occurs, renal excretion compensatorily increases^23–25^. In our study, patients with type 2 diabetes might have increased excretion of Gd-EOB-DTPA through the renal pathway. Consequently, the observation group's liver to kidney signal intensity score was altered. A recent study has shown that in patients with abnormal liver function (chronic liver disease and cirrhosis), the FLIS and its three sub-items strongly correlate with the liver Child–Pugh score^2^. In contrast, patients’ liver function was normal in our observation group. Perhaps type 2 diabetes was insufficient to alter all three sub-items of the FLIS in our patients. Although the liver to kidney signal intensity was changed in the diabetes patients of our study, there were no between-group differences in the other two parameters of the FLIS. For this reason, FLIS showed no statistically significant difference between the two groups.

The high interrater agreement for the FLIS, including each of the three measures (0.84–0.95), indicated that they were not affected by type 2 diabetes. Our results affirm the usefulness of the FLIS and agree with numerous prior studies^2,26,27^.

Our results should be considered within the context of several limitations. Firstly, because this study was retrospective in nature, it’s possible that there was a potential selection bias. Secondly, the patients were not sub-divided according to type 2 diabetes severity levels, given the relatively small size of our study cohort. However, we selected subjects with normal liver and renal function to minimize the influence of different severity of type 2 diabetes on the study process.

Although those drawbacks, our research shows that type 2 diabetes alters the imaging characteristics of the liver during the hepatobiliary phase. The signal intensity changes in the liver background during the hepatobiliary phase of patients with type 2 diabetes may adversely affect the diagnosis and detection of small hepatocellular carcinoma. It may also affect the radiologist's assessment of liver function. Further, well-powered studies are required to determine the effect of diabetes on Gd-EOB-DTPA-enhanced MRI.

## Data Availability

Raw data used in this study are available from the corresponding author upon reasonable request.

## Abbreviations

AUC: Area under the curve
95% CI: 95% confidence interval
FLIS: Functional liver imaging score
Gd-EOB-DTPA: Gadolinium ethoxybenzyl diethylenetriamine pentaacetic acid
ICCs: Intraclass correlation coefficients
LER: Liver enhancement ratio
LPC: Contrast between liver parenchyma and portal vein
MRI: Magnetic resonance imaging
OATPs: Organic anion-transporting polypeptides
ROCs: Receiver operating characteristic curves
